# Review of Case Reports on Adverse Events Related to Pre-workout Supplements Containing Synephrine

**DOI:** 10.1007/s12012-022-09777-z

**Published:** 2023-01-13

**Authors:** M. L. L. de Jonge, L. C. Kieviet, M. Sierts, L. B. Egberink, M. A. G. van der Heyden

**Affiliations:** 1 Honours Program CRU+, University Medical Center Utrecht, Heidelberglaan 100, 3584 CM Utrecht, The Netherlands; 2 Division of Heart & Lungs, Department of Medical Physiology, University Medical Center Utrecht, Yalelaan 50, 3584 CM Utrecht, The Netherlands

**Keywords:** Synephrine, Pre-workout, Ischaemic heart disease, Arrhythmias, Caffeine, Case reports

## Abstract

The use of pre-workout supplements has become increasingly popular, including the use of supplements containing synephrine. Synephrine might stimulate weight loss and improve sports performance by its proposed adrenergic properties. However, with its increasing popularity, numerous cases of adverse events related to synephrine use have been reported. This study provides a comprehensive overview and analysis of current case reports related to the supplemental use of synephrine. The scientific literature on cases of adverse events related to synephrine intake was collected through August 2021 using Pubmed and Google Scholar and subsequently reviewed and analysed. We obtained 30 case reports describing a total of 35 patients who suffered from medical complaints following use of synephrine-containing supplements. The patients most often presented with chest pain, palpitations, syncope and dizziness. Commonly raised diagnoses were ischaemic heart disease, cardiac arrhythmias and cerebrovascular disease. Five patients were left disabled or remained on medication at last follow-up. We here show an association between the use of pre-workout supplements containing synephrine and adverse events, mainly related to the cardiovascular system. However, we cannot exclude a role of possible confounding factors such as caffeine. Thus, the use of pre-workout supplements containing synephrine may lead to serious adverse health events, and therefore, caution is needed.

## Introduction

Over the past two decades, the use of pre-workout supplements has increased, mainly for the purpose of weight loss and/or improved sports performance. However, this use is not without risk, with supplements containing ephedrine alkaloids, also called ephedra, as an important example [1]. In 2004, the Food and Drug Administration (FDA) prohibited the sale of products containing ephedra in response to hundreds of cases of adverse health effects related to its use, including severe cardiovascular and neurologic events [1, 2]. Since then, the use of alternatives for ephedra increased, mainly by substituting ephedra for synephrine, which has a similar structure to ephedrine [3].

**Fig. 1:**
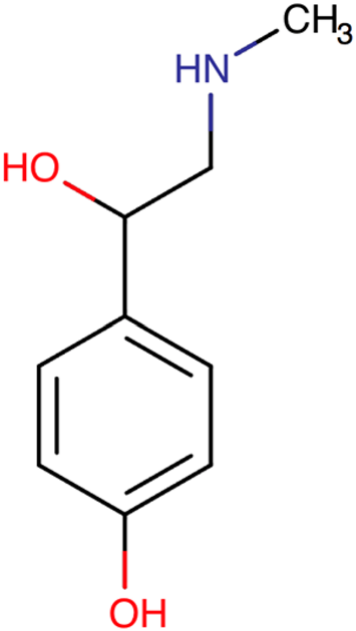
*p*-synephrine structure (drugbank DB09203)

Synephrine is a sympathetic stimulator and its isomer p-synephrine (Fig. 1) is present in high concentrations in *Citrus aurantium* (bitter orange) and other citrus fruits [4]. M-synephrine, a more potent isomer of synephrine, does not naturally occur in *C. aurantium*, but is sometimes added to *C. aurantium* supplements [5]. The desired effect of synephrine is caused by stimulation of beta-3 adrenoreceptors and thereby increasing lipolysis and metabolic rate [6]. However, synephrine intake might also lead to cardiovascular effects by the stimulation of additional adrenergic receptors (beta-1 and beta-2) [5]. It is thought that the combination of synephrine and caffeine may lead to synergistic effects in fat burning, but might also increase the risk of cardiovascular adverse effects [7].

Currently, most countries lack legislation on the permitted amount of synephrine in dietary supplements. Nevertheless, several regulatory authorities including those in the Netherlands have performed risk assessments on synephrine use and are considering banning the sale of synephrine in dietary supplements [8]. Despite the recognition of the possible adverse health effects of synephrine, legislation falls behind because of a lack of consistent data on a safe amount of synephrine use [8]. To date, toxicological data on synephrine are limited, especially concerning long-term intake [5]. The observed effects of synephrine intake obtained from mice and several human studies mainly consist of an increase in heart rate and blood pressure [5], but some studies do not report any negative effects and suggest that the daily use of synephrine is safe [9].

However, because of the amount of case reports related to the adverse effects on synephrine intake, further assessment appears to be essential to determine the safety of synephrine use. In this study, we sought to contribute to this knowledge, by providing an overview, comparison and analysis of the current case reports related to the supplemental use of synephrine. The symptoms, diagnosis, treatment and outcome of these case reports are analysed and discussed.

## Methods

### Data Collection

A literature search was conducted for case reports related to synephrine intake in August 2021 using the search engines Pubmed and Google Scholar. Firstly, the search term consisted of synephrine and its synonyms, including xenadrine, bitter orange, citrus aurantium, sour orange and oxedrine. These search terms were combined with adverse events or effects, safety, syndrome and filtered to include case reports only. This search yielded 28 results, of which seven were excluded for the reason that these articles did not focus on effects of synephrine use in particular. An additional number of two case reports were found through references of the initial 21 case reports. Furthermore, four case reports were found using only ‘synephrine case report’ as search term and one when using ‘citrus aurantium’ as the only search term. One synephrine case report was found when looking for ‘pre-workout induced’ case reports. Finally, searches were repeated in multiple languages, which yielded one additional case report in Polish, for a total of 30 case reports describing 35 subjects.

### Data Analysis

In order to minimise interobserver variability, all the case reports in this study were read and interpreted by at least two authors of the current paper. We consulted a native speaker concerning the article written in Polish. Statistical analysis was performed using SPSS® 28.0.1.0 (IBM®, USA). Categorical variables are presented as absolute values and percentages, continuous variables are expressed as mean ± standard deviation. Diagnoses and symptoms were categorised using the International Classification of Diseases system (ICD-10).

## Results

**Fig. 2:**
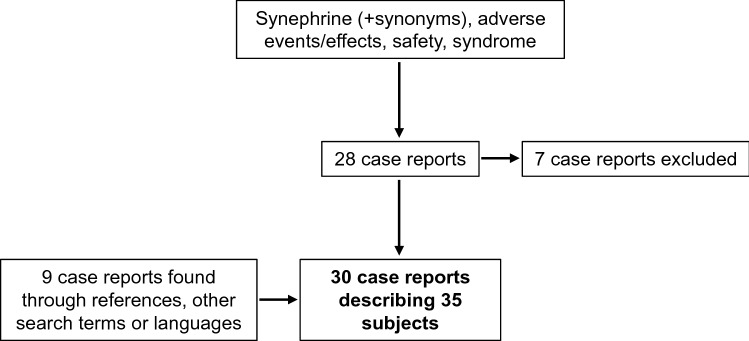
Flow diagram of data collection

Our search yielded 30 case reports describing a total of 35 patients who presented with adverse events related to the use of supplements containing synephrine (Fig. 2) [3, 10–38]. The case reports originated from eight different countries: United States (*n* = 21), South Korea (*n* = 1), Italy (*n* = 2), Poland (*n* = 1), Ecuador (*n* = 2), South Africa (*n* = 1), Australia (*n* = 1) and the Netherlands (*n* = 1). The case reports described patients with ages ranging from 16 to 57 years with a mean age of 32 ± 12 years (Table 1). Sixteen (50%) patients were male, and sixteen (50%) were female, whereas one case report did not mention age nor sex of its three patients. Many patients used synephrine-containing supplements for weight loss purposes (*n* = 18). The median duration of supplement usage, when mentioned, was 3 weeks (*n* = 28). The time between consumption of the synephrine-containing supplement and adverse effects varied in the studies.

**Table 1:** Baseline characteristics of study population

	All (*n* = 35)	Male (*n* = 16)	Female (*n* = 16)
Age (mean ± SD)	32 ± 12	31 ± 10	34 ± 13
Risk factors present			
Yes	10	6	4
No	10	4	6
Unknown	15	6	6
Purpose of use			
Weight loss	18	6	12
Exercise performance	5	3	2
Bodybuilding	3	3	0
Unknown	9	4	5
Mean dose synephrine, mg ± SD	41.9 ± 28.8	29.4 ± 16.3	56.9 ± 35.0
Mean duration of use (weeks (IQR))	3 (12)	8 (32)	2 (6)
Outcome			
Unknown	7	3	2
Full recovery	15	6	9
Lasting effects	5	4	1
Unknown	15	6	6

*SD* standard deviation, *mg* milligrams

Risk factors included smoking, alcohol use, high body mass index, hyperlipidaemia, family history for hypertension, heart murmur and eating disorder

### Risk Factors and Medication

A variety of possible risk factors were mentioned in the case reports (Table 1): smoking (*n* = 7, of which five current smokers and two ex-smokers), alcohol consumption (*n* = 2), high body mass index (above 25 kg/m^2^) (*n* = 6), hyperlipidemia (*n* = 1), positive family history for hypertension (*n* = 1), heart murmur (*n* = 1) and eating disorder (*n* = 1). The amount of smoking varied from five to sixty pack years. Five patients presented with multiple risk factors. On the other hand, ten patients were described to have no risk factors and in fifteen patients, the presence of risk factors was not reported.

In nineteen patients, the medication use was not mentioned in the case report. Seven patients did not use any medication. Of the patients using medication, one patient used aspirin as well as a cholesterol-lowering drug (fenofibrate). Two patients used levothyroxin for hypothyroidism; no recent change in dosage was mentioned.

### Symptoms

Seventeen patients complained of general signs or symptoms, including syncope (*n* = 6), headache (*n* = 3) and convulsions (*n* = 2) (Table 2). Symptoms of chest pain were mentioned eleven times, sometimes accompanied by vomiting, sweating, pain between shoulder blades or shortness of breath. Four patients experienced palpitations and three complained of dyspnoea. Twelve patients presented with symptoms involving cognition, e.g. dizziness (*n* = 6) and agitation (*n* = 3). Furthermore, two patients showed abnormalities of gait, two others had a tremor. Five patients showed skin symptoms such as urticaria. Three patients had abdominal symptoms including rectal bleeding (*n* = 2). Visual disturbances such as loss of vision were mentioned in three patients. Four patients presented with myalgia, other complaints regarding soft tissues were pain in limbs (*n* = 1), muscle weakness (*n* = 1) and arthralgia (*n* = 1). Two patients presented with symptoms of cerebral palsy, such as facial droop (*n* = 1) and hemiplegia (*n* = 1). Remaining symptoms included hyperventilation (*n* = 1), dysarthria (*n* = 1) and angioneurotic oedema (*n* = 1).

**Table 2:** Symptoms described in patients

Symptoms		Number of times mentioned
General symptoms and signs	Headache	3
	Malaise and fatigue	2
	Syncope and collapse	6
	Convulsions, not elsewhere classified (seizure)	2
	Hyperhidrosis/sweating	3
	Fever	1
Symptoms and signs involving cognition, perception, emotional state and behaviour	Dizziness and giddiness	6
	Amnesia	2
	Disorientation	1
	Symptoms involving emotional state	
	Agitation, restlessness, anxiety, irritability etc	3
Symptoms and signs involving the nervous and musculoskeletal system	Abnormalities of gait and mobility	2
	Tremor	2
Symptoms and signs involving the skin and subcutaneous tissue	Pallor	2
	Anaesthesia of skin	1
	Urticaria	2
Symptoms and signs involving the digestive system and abdomen	Diarrhoea	1
	Rectal bleeding	2
Symptoms and signs involving the circulatory and respiratory system	Chest pain	11
	Palpitations	4
	Dyspnoea	3
	Hyperventilation	1
Visual disturbances and blindness	Diplopia	1
	Loss of vision	1
	Scotoma	1
Soft tissue disorders	Myalgia	4
	Pain in limb	1
	Muscle weakness (generalised)	1
Symptoms and signs involving speech and voice	Dysarthria	1
Cerebral palsy and other paralytic syndromes	Facial droop	1
	Hemiplegia	1
Joint disorders	Arthralgia	1
Other	Angioneurotic oedema	1

All symptoms were categorised using the ICD-10 system

Eight patients presented with not previously diagnosed hypertension and four patients with tachycardia. Both blood pressure and heart rate were normal in twelve patients. Fourteen patients presented with ECG abnormalities, including but not limited to ST elevation (*n* = 5), QT prolongation (QTc 476–537 ms) (*n* = 3), T wave inversion (*n* = 2) and right bundle branch block (*n* = 2) (Table 3). Abnormalities on echocardiography were found in seven patients, of which a reduced ejection fraction (25–40%) found in five patients was the most common (Table 3). Twelve patients had elevated troponin levels (0.19–56 ng/mL).

**Table 3:** Results of ECG and echocardiography assessments

ECG abnormalities	Number of times mentioned	Echocardiography abnormalities	Number of times mentioned
QT prolongation	3	Low EF	5
T wave inversion	2	Wall motion abnormalities	3
ST elevation	5	Biventricular hypertrophy	1
ST depression	1	Left ventricular dilatation	1
Atrial rhythm	2	Tricuspid aortic valve with regurgitation	1
Right bundle branch block	2	Ascending aorta dissection	1
J-point elevation	2	Apical ballooning of left ventricle	1
Axis deviation	2		
Left ventricular hypertrophy	1		
"Borderline ST changes"	1		

*ECG* electrocardiography

### Diagnosis

Most patients were diagnosed with diseases of the circulatory system, including ischaemic heart disease (*n* = 10), cardiac arrhythmias (*n* = 4), cerebrovascular disease (*n* = 2) and aortic dissection (*n* = 1) (Table 4). Furthermore, other diagnoses included ischaemic colitis (*n* = 2), rhabdomyolysis (*n* = 1), renal artery occlusion (*n* = 1), mental and behavioural disorders (*n* = 2), syncope (*n* = 2), urticarial vasculitis (*n* = 2) and retinal artery occlusion (*n* = 1). The diagnosis of four patients was unknown.

**Table 4:** Diagnoses made in patients described in case reports

Category of diseases	Subcategory	Diagnosis	Number of times mentioned
Diseases of the circulatory system	Ischaemic heart disease	Acute coronary syndrome	8
		Variant angina	1
		Cardiac ischaemia, unspecified	1
	Cardiac arrhythmias	Ventricular fibrillation	2
		Tachycardia	1
		Bradycardia (and hypotension)	1
	Hypertensive disease	Hypertensive urgency	1
	Other forms of heart disease	Cardiomyopathy	2
	Diseases of arteries, arterioles and capillaries	Aortic dissection	1
	Cerebrovascular disease	Stroke	2
Diseases of the musculoskeletal system and connective tissue	Disorders of muscle	Rhabdomyolysis	1
Diseases of the digestive system	Vascular disorders of intestine	Ischaemic colitis	2
Diseases of the genitourinary system	Disorders of the kidney	Renal artery occlusion	1
Mental and behavioural disorder		Organic, including symptomatic mental disorders	1
		Acute poisoning by dietary supplement	1
Syncope and collapse		Syncope and collapse	2
Diseases of the skin and subcutaneous tissue		Urticarial vasculitis	2
Diseases of the eye and adnexa	Disorders of choroid and retina	Retinal artery occlusion	1
Unknown			4

All diagnoses were categorised using the ICD-10 system

### Dose and Duration of Use

In the eleven patients with a reported synephrine dose, the dose had a wide range (12 to 100 mg) (Table 1). One patient consumed a non-specified overdose according to the authors. The duration of use varied from days to multiple years. Four patients increased their intake of synephrine within a few days before symptoms started, one of those patients received the presumed overdose.

### Combination with Other Substances

The synephrine supplements used by the reported patients often contained various other substances. Frequently mentioned additional substances were caffeine (*n* = 19, *n* = 4 for Guarana, a herb containing a high dose of caffeine [39]), yohimbine (an indoline alkaloid, *n* = 9 [40]), deterenol combined with theophylline (*n* = 5) [35, 41] and beta-phenylethylamine (*n* = 5). Of all twenty patients diagnosed with diseases of the circulatory system, including ischaemic heart disease, cardiac arrhythmias and stroke, sixteen (80%) used a supplement containing synephrine combined with caffeine.

### Treatment

All patients received treatment based on their symptoms and work diagnosis. Some patients required more stringent treatment than others, for example, two patients required intubation and resuscitation or defibrillation. Three patients received oxygen therapy during their hospitalisation. Five patients underwent cardiac catheterisation, one patient underwent cardioversion, and one patient needed an aortic dissection repair. Two patients received coronary artery stents. Fourteen patients received aspirin or other anticoagulants. Most of the patients received pharmacological treatment, such as beta-blockers (*n* = 8), nitroglycerin or nitroprusside (*n* = 7), morphine or other analgesics (*n* = 3), antiplatelet or thrombolytics (*n* = 5), ACE-inhibitors (*n* = 4), ADP receptor inhibitors (*n* = 3) or statins (*n* = 3).

### Outcome

Twenty case reports mentioned a long-term outcome at an average follow-up duration of 23 ± 20 weeks. Most patients were without symptoms; however, five patients were left disabled or remained on medication at the last-mentioned follow-up (Table 5). Three of those patients had known risk factors for cardiovascular disease. Of the patients with known absence of risk factors, none were still taking medication or experiencing lasting effects at last follow-up.

**Table 5:** Patients with non-resolved outcome

Case report author [Ref]	Duration of follow-up (weeks)	Diagnosis	Outcome	Risk factors
Burke [10]	Unknown	Exercise-induced rhabdomyolysis and heat exhaustion (2 ×) complicated by acute renal failure and bilateral compartment syndrome	Permanent bilateral sensory and motor neurological deficits in both distal lower extremities	BMI 31
Elwood [37]	12	Transient partial central retinal artery occlusion with paracentral acute middle maculopathy	Mild, residual middle retina thinning consistent with chronic presentation of paracentral acute middle maculopathy	Unknown
Manivannan [20]	Unknown	ACS	Ejection fraction 39%, remained on anticoagulation	Unknown
Nykamp [23]	Unknown	Acute lateral wall MI	Cardiac rehabilitation programme for 5 months starting 3 months after event	Alcohol (socially), smoking (1,5 pack/day, since 18 years old), heart murmur, physical inactivity, (high caffeine intake)
Unnikrishnan [34]	Unknown	STEMI	Outpatient cardiology follow-up and advice to be maintained on dual antiplatelet therapy	BMI 28

*BMI* body mass index, *MI* myocardial infarction, *STEMI* ST-elevated myocardial infarction

No significant predicting variables for the outcome were found, but there was a tendency towards a better outcome in female patients. Out of ten female patients with a known outcome at last follow-up, nine had made a complete recovery compared to six out of ten male patients.

### Hypothesis

In all 30 case reports, the authors hypothesised the supplement as the cause of the adverse events observed in the described patients. Ten of these case reports mentioned synephrine as the specific cause for the events. In two cases, the authors reasoned that predisposing factors, such as a history of preeclampsia, were part of the explanation for the events in combination with the supplement use.

## Discussion

We reviewed 30 case reports describing 35 patients with adverse events related to the use of supplements containing synephrine. The most frequently mentioned symptoms were chest pain, palpitations, syncope and dizziness. Diagnoses were mainly related to the circulatory system, such as ischaemic heart disease, cardiac arrhythmias and cerebrovascular disease. Five patients were left disabled or remained on medication at last follow-up. A tendency towards a better outcome in female patients was observed but no explanation was found. Thus, this study demonstrates an association between synephrine intake and adverse, mainly cardiovascular, events.

Synephrine is thought to function as a sympathetic adrenergic agonist, stimulating both alpha and beta adrenoreceptors. It could potentially stimulate weight loss by stimulation of beta-3 adrenoceptors and thereby cause an increase in lipolysis, resting metabolic rate and energy expenditure. Moreover, synephrine is thought to cause effects in many more systems next to metabolism, including the cardiovascular system by stimulation of beta-1 and beta-2 receptors, causing an increase in heart rate and blood pressure [42, 43].

In line with the proposed adrenergic effects of synephrine, several human studies found that synephrine intake was correlated to weight loss [44], but the supplements assessed in these trials contained ephedrine besides synephrine, or results were not statistically significant [45]. However, synephrine was also found to potentially cause cardiovascular toxicity, by inducing effects such as ECG abnormalities and cardiac arrhythmias, and even death in animal studies [5]. As stated by Rossato et al., the observed increase in heart rate in human studies can cause a higher oxygen demand of cardiac myocytes, potentially leading to severe cardiac effects because of myocardial ischaemia [42]. It is important to note that available clinical data are limited to small short-term studies; no studies in which healthy volunteers are exposed to synephrine for a period longer than 60 days currently exist [5].

The results in this study contradict the claims of Stohs et al., who argue that the use of p-synephrine is safe and does not lead to adverse events, based on claims that there are several confounding factors that affect the conclusions in human studies including a medical history in the patients of obesity, smoking, physical inactivity and high caffeine intake, as well as the supplements not being taken as per their intended use. For these reasons, according to Stohs, such studies can therefore not be used properly to advise against the use of synephrine [9]. With regard to the claims of Stohs concerning the possible confounding influence of several other ingredients including caffeine in synephrine intake and observed adverse effects, in our study, it was unclear to what extent the effect of caffeine influenced our results. Many of the patients diagnosed with diseases of the circulatory system used a supplement containing synephrine as well as caffeine. Besides caffeine, the effect of the other various ingredients in the supplements such as yohimbine, deterenol and theophylline is also unknown and could play a role in the pathogenesis of the events leading to elevation of blood pressure [40] or cardiovascular toxicity [41, 46]. In conclusion, all potential confounders make it difficult to determine to what extent synephrine on itself has a role in the development of side effects. Nevertheless, it does not seem appropriate to state that the use of supplements containing synephrine is safe and without side effects, considering most supplements contain other active ingredients such as caffeine. Further research is needed to observe the effect of synephrine separately.

The strength of the current review lies in the minimisation of interobserver variability, for all case reports in this study have been read, interpreted and discussed by at least two authors until agreement was reached. A comprehensive search method using multiple search engines was used; despite this, search efforts yielded a relatively small number of cases, and analysis and comparisons were hampered by unreported data, as well as a large variety of patient characteristics and presentations. In particular, the dosage of synephrine was largely unknown; often, the supplement brand was mentioned but not the amount of synephrine it contained. None of the cases involved synephrine as the only ingredient, and supplementary ingredients were not always listed or described, leading to confounding as described above. Another limitation inherent to case report reviews is publication bias, skewing the results to more serious diagnoses as minor cases are not generally published.

To the best of our knowledge, we reviewed all available case reports on adverse events related to synephrine intake. As far as we know, this is the first review that provides an extensive overview of symptoms, diagnosis, treatment and outcome of patients that presented with adverse events related to the use of synephrine-containing supplements. Although we cannot rule out the influence of other confounding factors such as caffeine, this study showed that synephrine is able to induce serious health issues, mainly regarding the cardiovascular system, presumably because of adrenergic properties. Therefore, we believe that manufacturers of synephrine-containing supplements have the responsibility of warning its users of potential risks associated with synephrine intake, especially when combined with caffeine. Furthermore, physicians need to be aware of the possible side effects of synephrine intake and should continue to report on adverse events, including reporting the (presumed) dose of synephrine. We conclude that the use of synephrine may lead to serious adverse events, especially when supplements additionally contain caffeine, and therefore, caution is needed.
